# Analysis of ordinal data in clinical and experimental studies

**DOI:** 10.1590/1677-5449.200185

**Published:** 2020-11-11

**Authors:** Hélio Amante Miot

**Affiliations:** 1 Universidade Estadual Paulista – UNESP, Faculdade de Medicina de Botucatu, Departamento de Dermatologia e Radioterapia, Botucatu, SP, Brasil.

Certain phenomena are represented by qualitative data in which each category has a hierarchical relationship to the others (for example, educational level, functional class, phototype, and severity of symptoms). These data are known as ordinal data and should not be interpreted in the same manner as qualitative nominal data assigned to categories that are completely independent of each other (for example, marital status, gender, ABO typing, type of amputation, or type of aneurysm),^1^ but also cannot be interpreted in the same manner as quantitative data (for example, age, weight, blood pressure, and arterial flow), since there is not necessarily a fixed quantitative scale separating one category from another.^2^

Variables represented by ordinal data are very common in biomedical research and relate to clear concepts of a continuum of the intensity of effects, ordered according to a logical monotonic sequence, but not necessarily proportionally. These characteristics demand specific statistical techniques and if such techniques are not employed, analytical errors can occur that compromise the conclusions of analyses.^3^^,^^4^

Ordinal data provide less precise information than their quantitative alternatives, reducing analytical power. This has even more influence on the results if a study’s dependent variable is an ordinal variable. From a pragmatic perspective, all categorizations result in arbitrary reductionism and original data should therefore be collected as quantitative variables, which can be converted to ordinal data later. Moreover, collapsing categories together (for example, stage I vs. II vs. III + IV) or dichotomization of ordinal categories (for example, improvement vs. deterioration) penalizes information even further, making type II errors more likely.^5^^,^^6^ Thus, except for presentation of results or discussion of concepts, there is no clear analytical advantage to be gained from categorizing quantitative data and results that only attain significance through analysis of categorized data should be interpreted with caution, with awareness of the risk of type I error.^7^ Table 1 lists common ordinal variables used in clinical research and quantitative alternatives, which should be preferred in the exploratory phase of a study.

**Table 1:** Ordinal variables commonly used in clinical and experimental studies with their quantitative alternatives for data collection.

**Variable**	**Ordinal categories**	**Quantitative alternative**
Functional class	I, II, III, IV	N.A.
Educational level	Illiterate, primary, secondary, higher education	Full years in education
American Society of Anesthesiologists anesthetic risk	I, II, III, IV, V	N.A.
Age group	Children, adolescents, adults, seniors	Age in years
Cancer staging	I, II, III, IV	N.A.
Body composition	Underweight, healthy weight, overweight, obese, morbidly obese	Body mass index
Pallor	0, 1+, 2+, 3+, 4+	Hematocrit
Pulse amplitude	0, 1+, 2+, 3+, 4+	Plethysmography
Histopathological grading	0, 1+, 2+, 3+, 4+	Percentage of cells
Satisfaction	Very unsatisfied, fairly unsatisfied, neutral, satisfied, very satisfied	N.A.
Economic status	Classes A, B, C, D	Family income

N.A. = none available.

It is the researcher’s decision to present or analyze ordinal variables, whether because there is no quantitative equivalent (for example, cancer staging, satisfaction, relief from symptoms, level of amputation), because they offer a more appropriate representation of a concept related to a phenomenon (for example, surgical mortality in the morbidly obese compared with patients of a healthy weight), or even because the desired outcome is linked to an ordinal category (for example, achieving normal blood pressure is more important than a mean quantitative reduction of 10 mmHg in blood pressure).^8^

**Figure 1:**
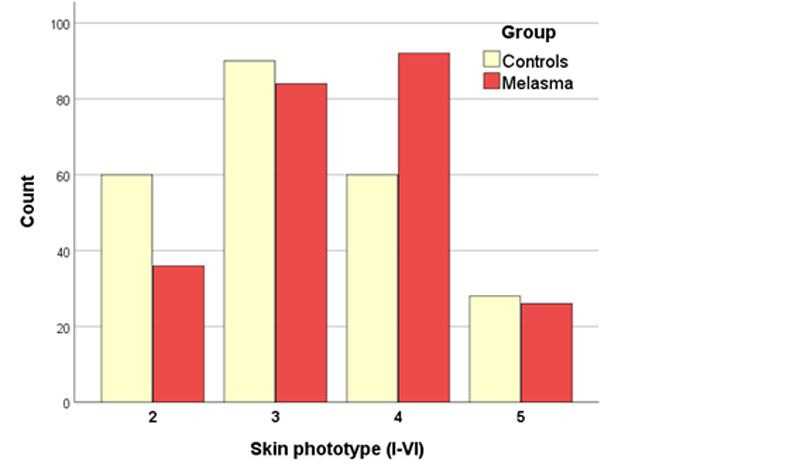
Graph illustrating frequencies of the ordinal variable skin phototype (I to VI) of melasma cases and controls from the Hospital das Clínicas de Botucatu (n = 238).^9^

During the ordinal data description step, researchers should be aware that, since the categories are independent, expressing results for a sample as means and standard deviations may not adequately describe them, whether because the distribution is not unimodal, because it is neither a normal distribution nor a symmetrical distribution, or even simply because the mean does not equate to any of the categories (for example, mean cancer stage = 2.5; mean pulse amplitude = 3.2 +). In such cases, it is preferable to describe the percentage frequencies of each category (for example, 10% stage I, 30% II, 40% III, and 20% IV), and illustrate them graphically using frequency plots (Figure 1).^9^^,^^10^ In cases in which there are several ordinal categories (≥ 5), the median should be given followed by the categories in which the quartiles fall (p25-p75), as long as the sample is unimodal, for example, a visual analog pain scale (VAS), or the American Society of Anesthesiologists anesthetic risk classification (ASA).^11^^-^15

Analysis of the difference between ordinal data distributed across two or more categories should prefer use of techniques that incorporate the direction of the effect inflicted by the ordering of the categories. Options for comparison of two ordinal categories are the chi-square test for trend (preferable for few ordinal categories) and the Mann-Whitney test; while the Kruskall-Wallis test can be used to compare more groups. Ordinal regression (logit or probit) can be used to compare several categories and also enables adjustment for covariates (such as sex, age, or comorbidities), offering the possibility of multivariate analysis of ordinal data.^4^^,^^13^^,^^15^^-^19

Table 2 illustrates an analysis of frequency by educational level, using these ordinal methods. Analyzing the same data with the chi-square test of independence (multinomial data) returns χ^2^ = 5.33 (p = 0.135), showing the importance of considering the ordinal nature of data in the analysis.

**Table 2:** Data from a hypothetical sample comparing frequency of a disease by educational level (n = 60).

	**Primary education (n = 18)**	**Secondary education (n = 24)**	**Higher education (n = 18)**	**Total (n = 60)**
**Sick – n (%)**	12 (67)	12 (50)	6 (33)	30 (50)
**Healthy – n (%)**	6 (33)	12 (50)	12 (67)	30 (50)

Chi-square test for trend (χ^2^ = 3.93; p = 0.046). Mann-Whitney test (U = 324.00; p = 0.047). Ordinal logistic regression (χ^2^ = 4.07; p = 0.043).

When the behavior of a quantitative variable is compared with ordinal categories (for example, age of students by social class), comparisons for normal and homoscedastic distributions should be made using analysis of variance (ANOVA) with linear contrast, which incorporates the ordinal nature of the categories and enables group trends to be inferred. For other conditions, its non-parametric alternative can be used: the Jonckheere-Terpstra test.^14^^,^^20^

Analysis of correlations that involve at least one variable with ordinal data should be conducted using the Spearman’s rho (ρ) or Kendall’s tau-b coefficients.^21^ Correlation between two ordinal variables with a small number of ordinal categories (< 5), as in quality of life questionnaire items, is a special case. In such cases, polychoric correlation should be preferred because it produces a less biased estimator. In turn, analyses of agreement can be performed using the weighted kappa test, which offers a similar estimate to the Intraclass Correlation Coefficient (for full agreement), or with the Kendall-W test.^22^^-^24

Longitudinal studies involving ordinal data can be analyzed using non-parametric models for dependent data (for example, the Wilcoxon and Friedman tests).^25^ However, when there are subgroups to be compared over time, temporal differences can be compared on the basis of changes to each category as a function of each observation (using, for example, the Mann-Whitney test or ordinal logistic regression) or, in a more sensitive manner, using multilevel models, such as generalized estimating equations or generalized linear mixed-effects models, weighted for ordinal distributions. These last two options demand supervision by an experienced statistics professional.^26^^-^29

Measurement instruments comprising items with ordinal scores (for example, quality of life surveys) should be assessed for dimensionality and can be more adequately analyzed using models based on item response theory for ordinal data.^30^^,^^31^

Finally, there is a certain degree of controversy with relation to exclusive use of ordinal statistical technique (non-parametric methods) for all cases, because of their lower statistical power compared to parametric techniques. Even using exact techniques (such as Monte Carlo methods, for example) for estimating p-values, non-parametric variants return more conservative results in terms of rejection of the null hypothesis.

Indeed, in unimodal and symmetrical ordinal distributions, as the sample size increases (for example, n > 30), the number of ordinal categories increases (for example, n ≥ 5), and where the intervals between categories are relatively constant (for example, age groups or seasons of the year), parametric statistical techniques offer adequate inferential performance for analysis of ordinal data. This argument is based on the central limit theorem^6^^,^^32^^,^^33^ and in the example in Table 2 (n = 60), Student’s *t* test results t = 2.03 (p = 0.046). However, because of the peculiar discontinuous and finite characteristics of ordinal values, the use of parametric techniques (which assume values that are continuous and infinite bilaterally) can increase type I error.^3^^,^^8^^,^^19^^,^^34^^,^^35^

The decision to use ordinal variables in a study demands detailed description in the methodology covering both the reasons why quantitative variables are categorized and the descriptive and analytical strategies adopted.^36^
