# Supplementary material for: Analysis of ordinal data in clinical and experimental studies
Source: J Vasc Bras. 2020 Nov 11;19:e20200185. doi: 10.1590/1677-5449.200185 (PMC8217997; doi:10.1590/1677-5449.200185)
Supplement: Translation

# Translation: Análise de dados ordinais em estudos clínicos e experimentais

**Authors:** Hélio Amante Miot

**Affiliations:** 1 Universidade Estadual Paulista – UNESP, Faculdade de Medicina de Botucatu, Departamento de Dermatologia e Radioterapiaz, Botucatu, SP, Brasil.

Há fenômenos que são representados por certos dados qualitativos em que cada categoria mantém uma dependência hierárquica em relação a outra (por exemplo, escolaridade, classe funcional, fototipo, gravidade dos sintomas). Esses dados são chamados ordinais e não devem ser interpretados como dados qualitativos nominais, que se caracterizam por categorias completamente independentes entre si (por exemplo, estado civil, gênero, tipagem ABO, tipo de amputação, tipo de aneurisma)^1^, tampouco como dados quantitativos (por exemplo, idade, peso, pressão arterial, fluxo arterial), já que não existe necessariamente uma escala quantitativa fixa que separa uma categoria de outra^2^.

As variáveis representadas por dados de natureza ordinal são muito comuns em pesquisa biomédica e retornam conceitos claros sobre um *continuum* de intensidade do efeito, ordenados segundo uma sequência lógica monotônica, mas não são necessariamente proporcionais. Por essa razão, suas características demandam técnicas estatísticas específicas, cuja inobservância leva a equívocos analíticos que podem prejudicar as conclusões^3^^,^^4^.

Dados ordinais apresentam menor precisão da informação do que suas alternativas quantitativas, reduzindo o poder da análise. Isso se torna ainda mais sensível para os resultados quando a variável ordinal é a variável dependente do estudo. De forma pragmática, toda categorização resulta de um reducionismo arbitrário; assim, os dados devem ser originalmente coletados como variáveis quantitativas, podendo se optar pela sua ordenação posterior. Além disso, a fusão de grupos (por exemplo, o estadiamento I vs. II vs. III + IV) ou a dicotomização dessas categorias ordenadas (por exemplo, melhora vs. piora) penitencia ainda mais a informação, favorecendo o erro tipo II^5^^,^^6^. Dessa forma, exceto para a apresentação dos resultados ou a discussão de conceitos, não há clara vantagem analítica na ordenação de dados quantitativos, e os resultados que mostram significância apenas pela análise dos dados categorizados devem ser interpretados com cautela sob o risco de erro tipo I^7^. A Tabela 1 apresenta as principais variáveis ordinais empregadas em pesquisa clínica, assim como suas alternativas quantitativas, preferidas na fase exploratória do estudo.

**Tabela 1:** Variáveis de natureza ordinal comumente utilizadas em estudos clínicos e experimentais e suas alternativas quantitativas para coleta dos dados.

**Variável**	**Categorias ordinais**	**Alternativa quantitativa**
Classe funcional	I, II, III, IV	N.D.
Escolaridade	Analfabeto, fundamental, médio, superior	Anos de estudo completos
Risco anestésico da *American Society of Anesthesiologists* (ASA)	I, II, III, IV, V	N.D.
Faixa etária	Infância, adolescência, adulto, idoso	Idade em anos
Estadiamento do câncer	I, II, III, IV	N.D.
Composição física	Magro, eutrófico, sobrepeso, obeso, obeso mórbido	Índice de massa corporal
Palidez	0, 1+, 2+, 3+, 4+	Hematócrito
Amplitude de pulso	0, 1+, 2+, 3+, 4+	Pletismografia
Marcação histopatológica	0, 1+, 2+, 3+, 4+	Percentual de células
Satisfação	Muito insatisfeito, um pouco insatisfeito, neutro, satisfeito, muito satisfeito	N.D.
*Status* econômico	Classe A, B, C, D	Renda familiar

N.D. = não disponível.

É critério do pesquisador representar ou analisar variáveis ordinais, seja porque não haja equivalente quantitativo (por exemplo, estadiamento do câncer, satisfação, alívio de sintomas, nível de amputação), porque representam mais adequadamente um conceito relacionado ao fenômeno (por exemplo, mortalidade em cirurgias de obesos mórbidos em comparação a eutróficos) ou mesmo porque o desfecho desejado está ligado a uma categoria ordinal (por exemplo, tornar-se normotenso é mais importante que uma redução quantitativa média de 10 mmHg da pressão arterial)^8^.

Na etapa de descrição de dados ordinais, deve-se atentar que, como as categorias são independentes, a representação da amostra como média e desvio padrão pode não permitir sua adequada caracterização, seja porque a distribuição não é unimodal, por não ter distribuição normal nem simétrica ou mesmo pelo ponto médio não representar nenhuma das categorias (por exemplo, estadiamento médio = 2,5; pulso médio = 3,2 cruzes). Dessa forma, deve-se preferir a descrição das frequências percentuais de cada categoria (por exemplo, 10% estadiamento I, 30% II, 40% III e 20% IV), e sua representação gráfica como diagrama de frequências (Figura 1)^9^^,^^10^. Em casos com numerosas categorias ordinais (≥ 5), deve-se optar pela mediana seguida pelas categorias que representam os quartis (p25-p75), desde que a amostra seja unimodal, por exemplo, escala visual analógica de dor (VAS), ou a classificação de risco anestésico da *American Society of Anesthesiologists* (ASA)^11^^-^15.

**Figura 1:** Diagrama de frequências da variável ordinal fototipo de pele (I a VI) de casos de melasma e controles provenientes do Hospital das Clínicas de Botucatu (n = 238)^9^.

A análise da diferença entre dados ordinais em duas ou mais categorias deve priorizar o uso de técnicas que incorporem a direção do efeito infligida pela ordenação das categorias. As opções para a comparação de duas categorias ordinais são o teste do qui-quadrado de tendência (preferível para poucas categorias ordinais) e o teste de Mann-Whitney; enquanto o teste de Kruskall-Wallis permite comparar mais grupos. A regressão ordinal (*logit* ou *probit*), além de possibilitar a comparação de várias categorias, permite o ajuste por covariáveis (como sexo, idade, comorbidades), possibilitando a análise multivariada de dados ordinais^4^^,^^13^^,^^15^^-^19.

A Tabela 2 apresenta uma análise de frequência segundo a escolaridade, de acordo com esses métodos ordinais. A análise desses mesmos dados pelo teste do qui-quadrado de independência (dados multinomiais) retorna um χ^2^ = 5,33 (p = 0,135), valorizando a importância de se considerar a ordenação na análise.

**Tabela 2:** Dados de uma amostra hipotética que compara a frequência de uma doença de acordo com o nível de escolaridade (n = 60).

	**Fundamental (n = 18)**	**Médio (n = 24)**	**Superior (n = 18)**	**Total (n = 60)**
**Doentes – n (%)**	12 (67)	12 (50)	6 (33)	30 (50)
**Não doentes – n (%)**	6 (33)	12 (50)	12 (67)	30 (50)

Qui-quadrado de tendência (χ^2^ = 3,93; p = 0,046). Teste de Mann-Whitney (U = 324,00; p = 0,047). Regressão logística ordinal (χ^2^ = 4,07; p = 0,043)

Quando se compara o comportamento de uma variável quantitativa de acordo com categorias ordinais (por exemplo, a idade dos alunos segundo a classe social), a comparação para distribuições normais e homocedásticas deve ser feita pelo teste de análise de variância (ANOVA) com contraste linear, que incorpora a ordenação das categorias e permite a inferência quanto à tendência dos grupos. Para as outras condições, pode ser utilizada sua alternativa não-paramétrica: o teste de Jonckheere-Terpstra^14^^,^^20^.

A análise de correlações que envolvam ao menos uma variável com dados ordinais deve ser realizada através do coeficiente rho (ρ) de Spearman ou de Kendall tau-b^21^. Um caso especial é a correlação entre duas variáveis ordinais com pequeno número de categorias ordinais (< 5), como acontece nos itens de questionários de qualidade de vida. Nesses casos, a correlação policórica deve ser preferida por produzir um estimador menos enviesado. Já as análises de concordância podem ser conduzidas pelo teste kappa com pesos, que oferece estimativa semelhante ao coeficiente de correlação intraclasse (CCI) para completa concordância, ou pelo teste Kendall-W^22^^-^24.

Estudos longitudinais que envolvam dados ordinais podem ser analisados a partir de modelos não-paramétricos para dados dependentes (por exemplo, testes de Wilcoxon e de Friedman)^25^. Porém, quando há subgrupos para serem comparados em função do tempo, as diferenças temporais podem ser comparadas quanto às mudanças de cada categoria em função de cada observação (por exemplo, teste de Mann-Whitney ou regressão logística ordinal) ou de forma mais sensível, utilizando modelos multiníveis como equações de estimativas generalizadas (GEE) ou modelos lineares generalizados de efeitos mistos, ponderados para distribuições ordinais. Esses últimos requerem a supervisão de um profissional estatístico experiente^26^^-^29.

Os instrumentos de medida compostos por itens com escores ordinais (por exemplo, inquéritos de qualidade de vida) devem ser avaliados quanto à dimensionalidade e podem ser analisados de forma mais adequada a partir de modelos que utilizem a teoria de resposta ao item (TRI) para dados ordinais^30^^,^^31^.

Finalmente, há certa controvérsia sobre o uso exclusivo de técnicas estatísticas ordinais (não-paramétricas) para todos os casos, visto o menor poder estatístico comparado às técnicas paramétricas. Mesmo com o uso de técnicas exatas (por exemplo, o método de Monte Carlo) para a estimativa do p-valor, as variantes não-paramétricas apresentam resultados mais conservadores quanto à rejeição da hipótese nula.

De fato, em distribuições ordinais unimodais e simétricas, à medida que a amostra se torna mais numerosa (por exemplo, n > 30), o número de categorias ordinais aumenta (por exemplo, n ≥ 5) e haja intervalos relativamente constantes entre as categorias (por exemplo, faixas etárias, estações do ano), as técnicas estatísticas paramétricas apresentam desempenho inferencial adequado para a análise de dados ordinais. Esse argumento se fundamenta no teorema do limite central^6^^,^^32^^,^^33^ e no exemplo da Tabela 2 (n = 60), o teste *t* de Student resulta em t = 2,03 (p = 0,046). Entretanto, devido à característica própria da descontinuidade e finitude dos valores ordinais, o uso de técnicas paramétricas (que os consideram valores contínuos e infinitos bilateralmente) pode levar ao inflacionamento do erro tipo I^3^^,^^8^^,^^19^^,^^34^^,^^35^.

A decisão de empregar variáveis ordinais na pesquisa exige descrição minuciosa na metodologia tanto dos motivos que levaram a categorizar variáveis quantitativas quanto das estratégias descritiva e analítica empregadas^36^.
